# Slipped-strand mispairing within a polycytidine tract in transcriptional regulator *mga* leads to M protein phase variation and Mga length polymorphism in Group A Streptococcus

**DOI:** 10.3389/fmicb.2023.1212149

**Published:** 2023-06-26

**Authors:** Benfang Lei, Tracey S. Hanks, Yunjuan Bao, Mengyao Liu

**Affiliations:** ^1^Department of Microbiology and Cell Biology, Montana State University, Bozeman, MT, United States; ^2^State Key Laboratory of Biocatalysis and Enzyme Engineering, School of Life Sciences, Hubei University, Wuhan, Hubei, China

**Keywords:** Group A Streptococcus, *Streptococcus pyogenes*, M protein, Mga, phase variation, slipped-strand mispairing

## Abstract

The M protein, a major virulence factor of Group A Streptococcus (GAS), is regulated by the multigene regulator Mga. An unexplained phenomena frequently occurring with *in vitro* genetic manipulation or culturing of M1T1 GAS strains is the loss of M protein production. This study was aimed at elucidating the basis for the loss of M protein production. The majority of M protein-negative (M^−^) variants had one C deletion at a tract of 8 cytidines starting at base 1,571 of the M1 *mga* gene, which is designated as c.1571C[8]. The C deletion led to a c.1571C[7] *mga* variant that has an open reading frame shift and encodes a Mga-M protein fusion protein. Transformation with a plasmid containing wild-type *mga* restored the production of the M protein in the c.1571C[7] *mga* variant. Isolates producing M protein (M^+^) were recovered following growth of the c.1571C[7] M protein-negative variant subcutaneously in mice. The majority of the recovered isolates with reestablished M protein production had reverted back from c.1571C[7] to c.1571C[8] tract and some M^+^ isolates lost another C in the c.1571C[7] tract, leading to a c.1571C[6] variant that encodes a functional Mga with 13 extra amino acid residues at the C-terminus compared with wild-type Mga. The nonfunctional c.1571C[7] and functional c.1571C[6] variants are present in M1, M12, M14, and M23 strains in NCBI genome databases, and a G-to-A nonsense mutation at base 1,657 of M12 c.1574C[7] *mga* leads to a functional c.1574C[7]/1657A *mga* variant and is common in clinical M12 isolates. The numbers of the C repeats in this polycytidine tract and the polymorphism at base 1,657 lead to polymorphism in the size of Mga among clinical isolates. These findings demonstrate the slipped-strand mispairing within the c.1574C[8] tract of *mga* as a reversible switch controlling M protein production phase variation in multiple GAS common M types.

## Introduction

*Streptococcus pyogenes* or Group A Streptococcus (GAS) is a major human pathogen that commonly causes pharyngitis and skin infection (Carapetis et al., 2005). GAS can occasionally cause severe invasive infections, such as necrotizing fasciitis, pneumonia, and streptococcal toxic shock syndrome (Nelson et al., 2016). GAS produces many extracellular virulence factors to mediate its pathogenesis (Cunningham, 2008). The M protein is a coiled-coil α-helical surface protein that is covalently linked to the peptidoglycan of GAS (McNamara et al., 2008). The M protein is a major virulence factor and specifically acquires host proteins, such as fibrinogen and complement factor H, to block deposition of antibody and complement C3b and C3bi opsonins, preventing phagocytosis of GAS by neutrophils (Fischetti et al., 1988; Sandin et al., 2006).

The M protein gene, *emm*, is one of several genes with the highest expression in GAS at the exponential growth phage in nutrient-rich medium. The transcription of *emm* is regulated by the transcription activator Mga (for multigene regulator in GAS and formerly known as Mry) (Caparon and Scott, 1987; Ribardo and McIver, 2006). Mga directly activates *emm* and several other virulence genes (McIver et al., 1995). Expression of M protein in M12 type GAS exhibits phase variation, transiting from M protein-positive (M^+^) to M protein-negative (M^−^) phenotype during culture *in vitro* (Simpson and Cleary, 1987). Similarly, the loss of M protein production and down-regulation of *emm* transcription in some M1 type GAS strains was observed fairly frequently in conjunction with genetic manipulations for the generation of gene deletion mutants unrelated to the *emm* gene (Zhou et al., 2013). In as much as the loss of M protein production and down-regulation of *emm* transcription occurred independent of the gene being deleted, presumably it was caused by spontaneous secondary mutation(s) affecting M protein production but unrelated to the gene being deleted (Zhou et al., 2013). In both the M12 and M1 genetic backgrounds, the specific genetic change(s) underlying the loss of M protein production observed in these studies was not determined.

Phase variation in general refers to a reversible on/off switch for control of expression of one or more proteins between individual cells of a clonal population (van der Woude, 2011). One mechanism of phase variation is slipped-strand mispairing that classically affects DNA regions with direct repeats of 1–4 bases, with reversible genetic changes stemming from the misalignment of the repeat sequences of the mother and daughter strands during replication (Levinson and Gutman, 1987; Henderson et al., 1999; Viguera et al., 2001). Slipped-strand mispairing results in decrease or increase of the number of repeats in the newly synthesized DNA and can lead to altered gene expression at either the transcriptional or translational level (Stern et al., 1986; Stibitz et al., 1989; Lukomski et al., 2001; Rasmussen and Björck, 2001; Torres-Cruz and van der Woude, 2003). The *mga* sequences fall into two major clusters that display 25 to 30% divergence (Bessen et al., 2005). The *mga* genes in cluster 1 (*mga*-1), but not *mga*-2 cluster, have a tract of 8 continuous cytidine bases (polycytidine tract) starting at base 1,574, which is referred as c.1574C[8] according to the standard mutation nomenclature (Ogino et al., 2007). This report presents evidence that slipped-strand mispairing within the c.1571C[8] tract of *mga* functions as a switch for the phase variation of M protein and occurs in patients.

## Materials and methods

### Bacterial strains and media

Sequenced M1 strains SF370 (Ferretti et al., 2001), MGAS2221 (Sumby et al., 2006), MGAS5005 (Sumby et al., 2006), and 5448 (Walker et al., 2007), and M3 strain MGAS315 (Beres et al., 2002) were used in this study. These GAS strains and their derivatives were grown in Todd-Hewitt broth supplemented with 0.2% yeast extract (THY) at 37°C in 5% CO_2_. Tryptose agar with 5% sheep blood and THY agar were used as solid media.

### *In vitro* culture condition that led to GAS variants with loss of M protein production

MGAS2221 was cultured in THY starting with an optical density at 600 nm (OD_600_) of 0.05 at 37°C in 5% CO_2_. The continued culture was done by adding 1 mL of the prior culture to 11 mL of THY and grown for 12 h. This serial passage continuous culturing process was repeated for 15 cycels. Following the 15th passage bacteria were harvested at an OD_600_ of 0.3, diluted and plated on THY agar. Randomly picked colonies were streaked on plates, grown to mid-exponential growth, and stored frozen until analysis for detection of the M protein by Western blotting analysis. Gene deletion mutants of SF370, MGAS2221, MGAS5005, and MGAS315 were obtained in a two-step in-frame gene deletion procedure as described (Zhu et al., 2009). The step in which mutants might lose M protein production was the growth of isolates from the first crossover in THY for >8 passages (one passage = 0.05 to 0.7 in OD_600_) that allowed the second crossover event to occur for the generation of gene deletion mutants.

### Cell surface M protein detection by Western blotting

The cell surface M protein of M1 GAS isolates was detected by Western blotting, as previously reported (Zhou et al., 2013). Briefly, bacteria in 10 mL culture with an OD_600_ of 0.2 were harvested by centrifugation, suspended in 0.2 mL phosphate-buffered saline (PBS), and digested with 10 μg PlyC (Nelson et al., 2006) at room temperature for 1 h. The supernatants containing the released cell wall proteins were diluted with 1:60 1x SDS-PAGE loading buffer, boiled, and 10 μL of each sample was resolved by SDS-PAGE. Proteins were transferred from SDS-PAGE gel to nitrocellulose membrane (Immobilon-NC, Millipore Corporation) with Towbin transfer buffer using a Trans-Blot Semi-Dry Transfer Cell (Bio-Rad Laboratories) at 15 V for 40 min. The membrane was blocked with 3% bovine serum albumin–0.1% Tween 20 in 20 mM Tris–HCl, pH 8.0, for 1 h and incubated for 1 h with 1:2000 anti-M1 antisera that was kindly provided by Dr. James Dale at University of Tennessee Health Science Center. The membrane was then rinsed twice and washed three times for 15 min with 0.1% Tween 20 in PBS. The membrane was incubated with goat anti-rabbit IgG (heavy + light chain) horseradish peroxidase (HRP) conjugate secondary antibodies for 1 h and rinsed and washed as described above. Antigen–antibody reactivity was visualized by enhanced chemiluminescence. Western blots usually shows two bands for M protein and the higher MW band is M protein with attached peptidoglycan fragments.

### Quantitative RT-PCR analysis

Data for *emm* mRNA levels in Supplementary Table S1 were associated with our previous publications (Zhu et al., 2009; Liu et al., 2012; Li et al., 2013; Zhou et al., 2013; Liu et al., 2015; Stetzner et al., 2015; Feng et al., 2017). Levels of *emm* and *gyrA* (control) mRNA were measured by using TaqMan quantitative RT-PCR assays with specific probes and primers as reported previously (Zhou et al., 2013) or using the All-in-One SYBR qPCR mix from GeneCopoeia (Stetzner et al., 2015). The transcription data for MGAS2221, GAS1806, M405, M406, and M497 were measured using the TaqMan quantitative RT-PCR as described by Zhou et al. (2013). The data were for early exponential growth phase at optical density 0.2 of GAS in THY. Control reactions that did not contain reverse transcriptase revealed no contamination of genomic DNA in any RNA sample. All RNA samples were assayed in triplicate, and the levels of *emm* mRNA were compared using the ΔΔCT method with normalization to the mRNA levels of the *gyrA* gene and presented with normalization to that of corresponding wild-type or control strain.

### Complementation of M^-^ variants with *mga*

The *mga* gene of MGAS2221 was cloned into pDCBB-RFA (Liu et al., 2013) with the Gateway Cloning Technology according to the manufacturer’s manual. pDCBB (Treviño et al., 2009) was modified by inserting the blunt-ended reading frame cassette A (RFA) into pDCBB at the EcoRV site, resulting in pDCBB-RFA (Liu et al., 2013). The *mga* gene was PCR amplified using Phusion DNA polymerase (New England BioLabs, Ipswich, MA) and the primers 5′- GGGGACAAGTTTGTACAAAAAAGCAGGCTagaagggtatacaaggtaatg-3′ and 5’-GGGGACCACTTTGTACAAGAAAGCTGGGTgtttttgagttgctacagtta-3′. The sequences in the capital letters were *attB* sequences for the BP clonase reaction. The PCR product was cloned into the donor vector pDONR221 using the BP clonase, yielding pDONR221-*mga*. The *mga gene* in pDONR221 was transferred into pDCBB-RFA by the LR clonase, yielding pDCBB-*mga*. pDCBB-*mga* or vector control pDCBB was introduced into M^-^ variants *via* electroporation.

### DNA sequencing

DNA sequencing of amplified PCR products was performed using the BigDye Terminator v3.1 Cycle Sequencing Kit and an Applied Biosystems 3130 genetic analyzer. Sequence data were analyzed using the software Sequencer 5.1 from the Gene Codes Corporation. To sequence the whole *mga* gene, a 2,454-bp fragment containing *mga* was amplified using Phusion DNA polymerase and the paired primers 5′-GTTGTACCATAACAGTCAAAC-3′/5′-TTTCAAGTTCTTCAGCTCTC-3′. The primers used for sequencing were the two PCR primers and additional primers, 5′-AACGAATCAAGTTAACTGAGC-3′, 5′-TCCTAAACTTAAAGAACTGTG-3′, 5′-TGTCACGATCACATCATACTG-3′, and 5′-TTTAACAGTGTTGGTAATTTC-3′. To sequence the c.1574C[8] region of M1 and M1T1 GAS isolates, a 469-bp fragment was amplified and sequenced using the primers 5′-TTTAAACATCAGCTTTGCAGA-3′ and 5′-TCTTCTATAACTTCCCTAGGA-3′. To sequence the c.1592C[8] region of M3 GAS isolates, a 457-bp fragment was amplified and sequenced using the primers 5′–TTTTTAAACATCAGCTCTGCAGA–3′ and 5′–CATTAACACTCCTAGCATCTG–3′.

### M^+^ isolates from M^-^ variants in subcutaneous infection of mice

M^-^ variants were grown in THY, harvested at the mid-exponential growth phase (OD_600_ of 0.4) and washed three times with and resuspended in pyrogen-free Dulbecco’s phosphate-buffered saline (DPBS). Five 5-weeks-old female C57BL/6 J mice were injected subcutaneously with 0.2 mL of GAS suspension in DPBS with an OD_600_ of 0.8. The mice were euthanized with CO_2_ at day 4 after inoculation. Skin infection sites were collected and homogenized in DPBS using a Kontes pestle, and plated at appropriate dilutions. Randomly picked 70 colonies were grown and analyzed for detection of M protein production using the western blotting analysis as described above. The mouse experimental procedures were carried out in strict accordance with the recommendations in the *Guide for the Care and Use of Laboratory Animals* of the National Research Council (2011).

### Analyses of *mga* gene and Mga protein sequences from genome databases

The DNA sequences of the *mga* gene and its protein sequences were analyzed for M1, M3, M12, M14, M23, and M49. Complete and incomplete GAS genomes as of October 10, 2019 were downloaded from the NCBI GenBank database.^1^ M type of GAS strains was determined by aligning their sequences with the *emm* sequences in an *emm* genotype database.^2^ Because there were no polymorphisms in the length of Mga-2 of M49 GAS, we just listed all of 158 M1, 106 M3, 106 M12, 6 M14, and 2 M23 strains that were available at the time for analysis (Supplementary Tables S2–S4 and Table 1). The nucleotides of *mga* and *emm* in each genome were extracted by mapping to the *mga* and *emm* locus using blast (Camacho et al., 2009). Multiple sequence alignments of *mga* DNA sequences and Mga protein sequences were performed using ClustalO (Sievers et al., 2011).

**Table 1:** Polymorphism in polycytidine tract of *mga* and Mga length of M14 and M23 GAS.

Strain	M type	Polycytidine tract	AA No. of Mga
NS501	M14	c.1574C[8]	530
NS506	M14	c.1574C[8]	530
33042V1T1	M14	c.1574C[8]	530
NS4985	M14	c.1574C[8]	530
NCTC8199	M14	c.1574C[6]	543
HSC5	M14	c.1574C[6]	543
N23ND	M23	c.1555C[5]/c.1573[9]	530
NCTC4001	M23	c.1555C[6]/c.1574C[6]	543

## Results

### M protein production is frequently lost during genetic manipulation for generation of GAS gene deletion mutants

**Figure 1:**
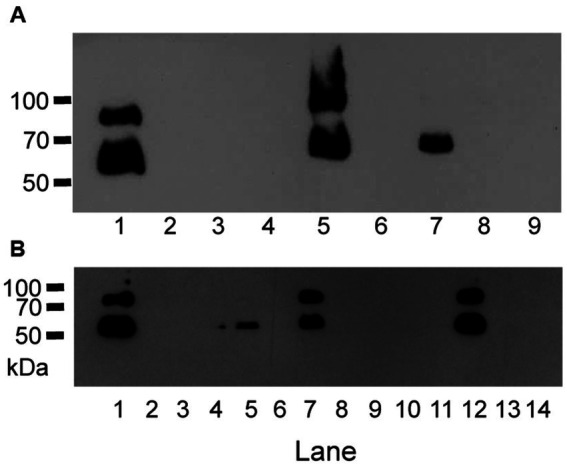
Anti-M protein western blots show GAS variants with loss of M protein production. **(A)** Anti-M protein blot of MGAS2221, MGAS5005 and SF370 and their gene deletion mutants. Lanes: 1, MGAS2221; 2, GAS1213 (MGAS2221Δ*spyCEP*); 3, GAS1473 (MGAS2221ΔsclA); 4, GAS1498 (MGAS2221Δ*spy1118*); 5, MGAS5005; 6, GAS1133 (MGAS5005Δ*sse*); 7, SF370; 8, GAS827 (SF370Δ*covS*); and 9, GAS1458 (SF370Δ*sclA*). **(B)** Anti-M protein blot of MGAS2221 (lane 1) and its derivative isolates from *in vitro* culture after 15 passages (lanes 2 to 14).

Reverse genetic analysis is a commonly used approach to define the function and phenotype of target genes. We use a two-step strategy to delete genes in-frame in GAS (Zhu et al., 2009). A suicide plasmid is constructed to contain the upstream and downstream flanking fragments of target gene that are joined to each other. The first step involves the first homologous crossover between one flanking fragment in the suicide plasmid and GAS chromosome, yielding chloramphenicol-resistant merodiploid transformants. The second step passes one transformant in liquid THY medium or on THY agar plate without chloramphenicol selection for more than 8 times to allow the second crossover between the other flanking fragment of the integrated suicide plasmid and its homologous sequence of GAS chromosome, resulting in gene deletion mutants. During genetic manipulation or any process that entails bacterial DNA replication, spontaneous random mutations can occur. To rule out the presence of a potential spurious mutation grossly altering the expression of known major virulence factors, western blotting analysis and/or qRT-PCR analyses were performed to check M protein production and the expression of M protein gene, *hasA*, *spyCEP*, and *sse* genes of gene deletion mutants. We have previously shown that genetic manipulation of GAS to construct gene deletion mutants can independently of the gene being targeted lead to the spontaneous loss of *emm* gene expression and M protein production (Zhou et al., 2013). To evaluate if loss of M protein production during such processes is a general phenomenon common to many GAS strains of M1 and other M types, we analyzed gene deletion mutants of M1 strains SF370, MGAS2221, MGAS5005, and 5,448 by the western blotting analysis and gene deletion mutants of M3 strain MGAS315. As shown in Figure 1, independent of the gene targeted for deletion, gene deletion mutants of M1 GAS strains MGAS2221, MGAS5005, and SF370 could lose M protein production. In total, 43 of 92 gene deletion mutants of M1 GAS strains MGAS5005, MGAS2221, 5448, and SF370 lost M protein production (Supplementary Table S1). As listed in Supplementary Table S1, 13 M^+^ and 11 M^-^ gene deletion mutants of them had relative mRNA levels of the *emm* gene that were obtained for our previous publications (Zhu et al., 2009; Liu et al., 2012; Li et al., 2013; Zhou et al., 2013; Liu et al., 2015; Stetzner et al., 2015; Feng et al., 2017). The *emm* mRNA levels in 9 of the 10 M^-^ gene deletion mutants were 1 to 5.5% of those in their parent strains whereas 1 M^−^, GAS1778 (5448Δsda1), and all the 11 M^+^ deletion mutants has similar levels of *emm* mRNA with those in their parent strain. Two of 9 gene deletion mutants of M3 strains MGAS315, GAS1191 and GAS1028, down-regulated *emm3* transcription by about 99%, and western blotting analysis was not performed for the M3 gene deletion mutants because anti-M3 protein antibody was not available (Supplementary Table S1).

The second crossover to achieve gene deletion occurred during growing first-crossover tranconjugants in THY medium or on THY agar plates. The 44 M^-^ gene deletion mutants were all among 70 mutants that were obtained by using THY medium for the second crossover whereas all 31 gene deletion mutants that were obtained by using THY agar were all positive in M protein production (Supplementary Table S1). These data suggest that M^-^ variants have an advantage over M^+^ GAS to survive in THY.

### M^-^ variants of MGAS2221 arise during culturing

To determine whether M^-^ variants of M1 GAS arise during culture *ex-vivo* in THY, strain MGAS2221 was passed in THY twice a day for 7 days, randomly chosen colonies after the last passage were analyzed for M protein production using western blotting analysis. Fifty colonies from the MGAS2221 culture after 14 passes were tested for M protein production, and 30 of them had no detectable M protein by Western blotting analysis. Figure 1B shows a representative Western blot in the analysis. Thus, M^−^ M1 GAS variants arise during *in vitro* growth, a phenomenon similar to that described for M12 GAS strains (Simpson and Cleary, 1987).

### Slipped-strand mispairing within the c.1571C[8] tract of *mga* causes loss of M protein in M1 GAS

**Figure 2:**
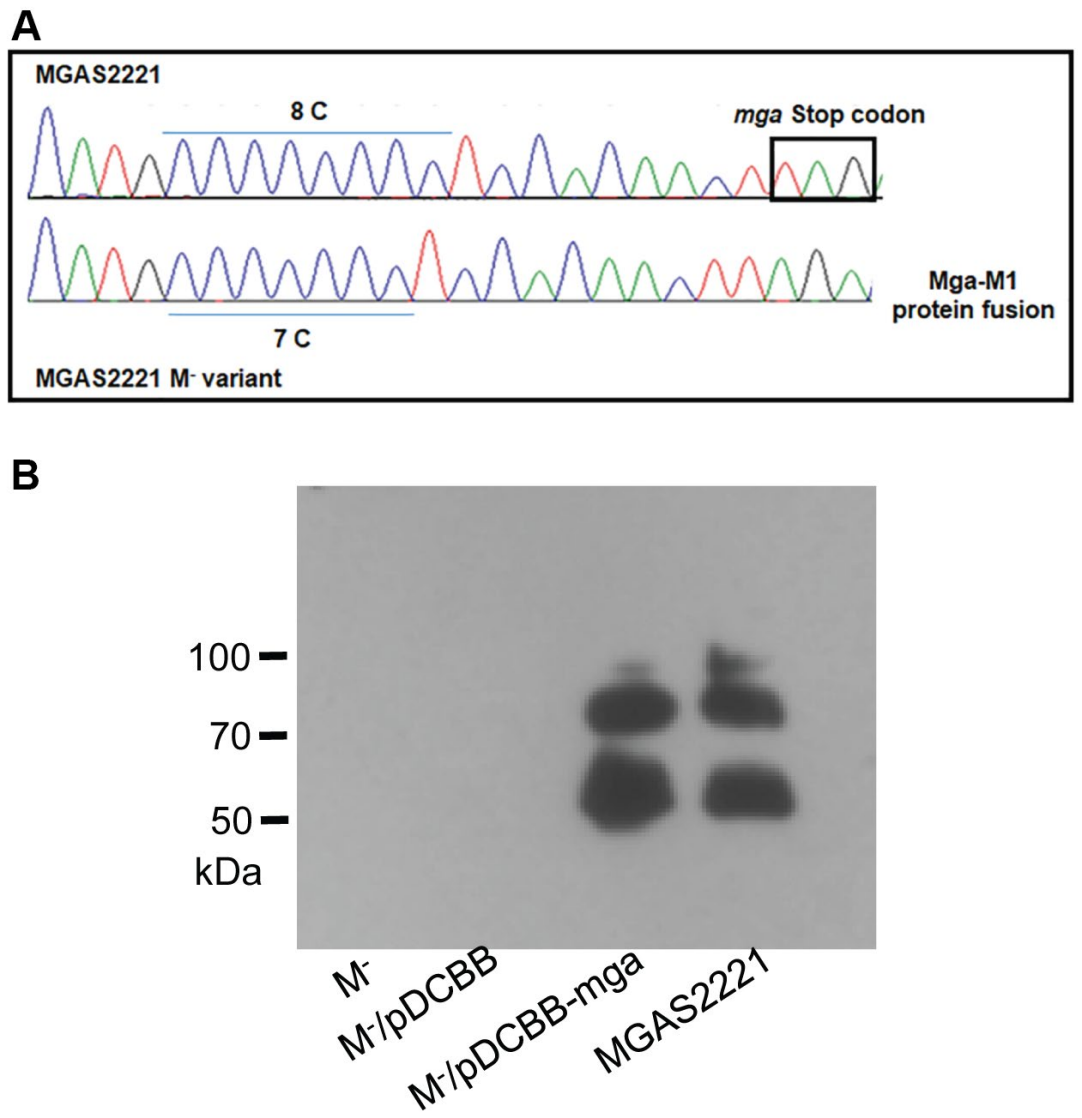
Slipped-strand mispairing within the c.1571C[8] polycytidine tract of *mga* turns off M protein production. **(A)** DNA sequencing chromatogram shows 8\u00B0C repeats of the polycytidine tract near the 3′ end of *mga* in MGAS2221 but 7\u00B0C repeats of the tract in its M^-^ variant (GAS1285) that led to a Mga-M1 protein fusion protein. **(B)** In trans complementation of the M^-^ variant with pDCBB-mga but not pDCBB (vector control) restored M protein production.

Most of 10 M^-^ variants with *emm* transcript data had dramatic downregulation in transcription of the *emm* gene (Supplementary Table S1). Since Mga is the primary regulator of *emm* expression any polymorphism in the *mga* gene or the promoter region of *mga* and/or *emm* that reduces the capacity of Mga to activate *emm* transcription could contribute to loss of the M protein expression. To look for such polymorphisms, a PCR amplicon containing the *mga* gene and its 379-bp upstream and 470-bp downstream sequences from one M^−^ variant and its parent strain MGAS2221 were sequenced by the Sanger DNA sequencing. A single C deletion was found in the polycytidine tract that starts at base 1,571 and has 8 repeats of C near the end of the *mga* gene of M1 GAS (Figure 2A). This polycytidine tract of wild-type *mga* is designated as c.1574C[8] in which 1,574 and 8 represent the starting base of the polycytidine tract and the number of the C repeats, respectively. The 1C deletion in the c.1571C[8] tract leads to the c.1571C[7] *mga* variant. The c.1571C[7] *mga* variant gene in M1 GAS has an altered reading frame of the *mga* gene, and the mutated *mga* gene is read through the intergenic region between the *mga* and *emm*1 genes and the *emm*1 gene, leading to a protein variant that contains the amino acid sequence of Mga, 62 amino acid residues encoded by the intergenic sequence, and M1 Protein. Transformation of the c.1571C[7] *mga* variant with plasmid pDCBB-*mga*, but not the plasmid vector control, restored the M protein production (Figure 2B).

To determine whether the c.1571C[7] *mga* variant was prevalent among our M^−^ strains, a 820-bp DNA fragment covering 349-bp of 3′ part of *mga*, 184-bp *mga*/*emm* intergenic region, and 287-bp of the 5′ part of *emm* was sequenced for 37 gene deletion mutants with diminished M protein production. The results are presented in Supplementary Table S1. 37 of the 43 M^−^ mutants of M1 strains MGAS2221, MGAS5005, 5,448, or SF370 had the c.1571C[7] *mga* variant whereas 6 mutants had the c.1574C[8] tract of *mga*. One of the 5 M^-^ mutants with the c.1571C[8] *mga*, GAS1099, had A-to-G mutation at base 74 upstream of *emm* that is referred as -74A > G of *emm*. This mutation changed the-10 box TACAAT of the *emm* promoter to TGCAAT and had reduction of *emm* mRNA by 97.9%. The whole *mga* gene of the 5 remaining M^-^ variants with c.1571C[8] of *mga* was sequenced. GAS874 had missense mutation of A to C at base 292, c.292A > C, leading to the threonine-to-proline mutation at residue 98 of the Mga protein, Mga T98P, and had reduction of *emm* mRNA levels by 94.5%. The other 4 M^-^ variants, GAS1499, GAS1081, GAS1185, and GAS1778, had the wild-type Mga and were not further characterized. The *mga* gene of 10 M^-^ variants from *in vitro* MGAS2221 culture were also sequenced. Eight of them had c.1571C[7]; isolate 499 had *mga* c.866A > C missense mutation that led to Mga T289P; and isolate 521 had wt c.1571C[8] *mga* (Table 2). Thus, the conversion of *wt* c.1571C[8] *mga* into the c.1571C[7] variant was a common cause for M^-^ variants arisen during genetic manipulation and simple *in vitro* culturing of M1 GAS strains.

**Table 2:** Polymorphism of *mga* in M protein-negative isolates of cultured MGAS2221.

Isolate No.	*mga* polycytidine tract	Other mutation
GAS1806	c.1571C[7]	
499	c.1571C[8]	*mga* c.866A > C Mga T289P
503	c.1571C[7]	
506	c.1571C[7]	
520	c.1571C[7]	
521	c.1571C[8]	
523	c.1571C[7]	
525	c.1571C[7]	
535	c.1571C[7]	
540	c.1571C[7]	

The *mga* gene of M3 GAS strains also has the polycytidine tract starting at base 1,592 that is referred as c.1592C[8]. Two gene deletion mutants of *emm*3 GAS strain MGAS315, GAS1191 and GAS1028, had reduction of *emm* mRNA levels by 99% and 1C deletion in the c.1592C[8] tract of *mga* (Supplementary Table S1). This 1-C deletion in the c.1592C[8] tract did not lead to the Mga-M3 fusion protein but led to an Mga variant with 52 extra amino residues in comparison with the wild-type Mga protein of M3 GAS. Unlike the M1 c1571C[7] *mga* variant, the c.1592C[7] *mga* variant of M3 GAS does not encode Mga-M protein fusion. This difference is due to the deletion of G at base 148 of the intergenic sequence between the *mga* and *emm* genes in M3 GAS in comparison with M1 GAS (Supplementary Figure S1).

### M^+^ isolates from M^-^ strains with c.1571C[7] *mga* variant in mice

**Figure 3:**
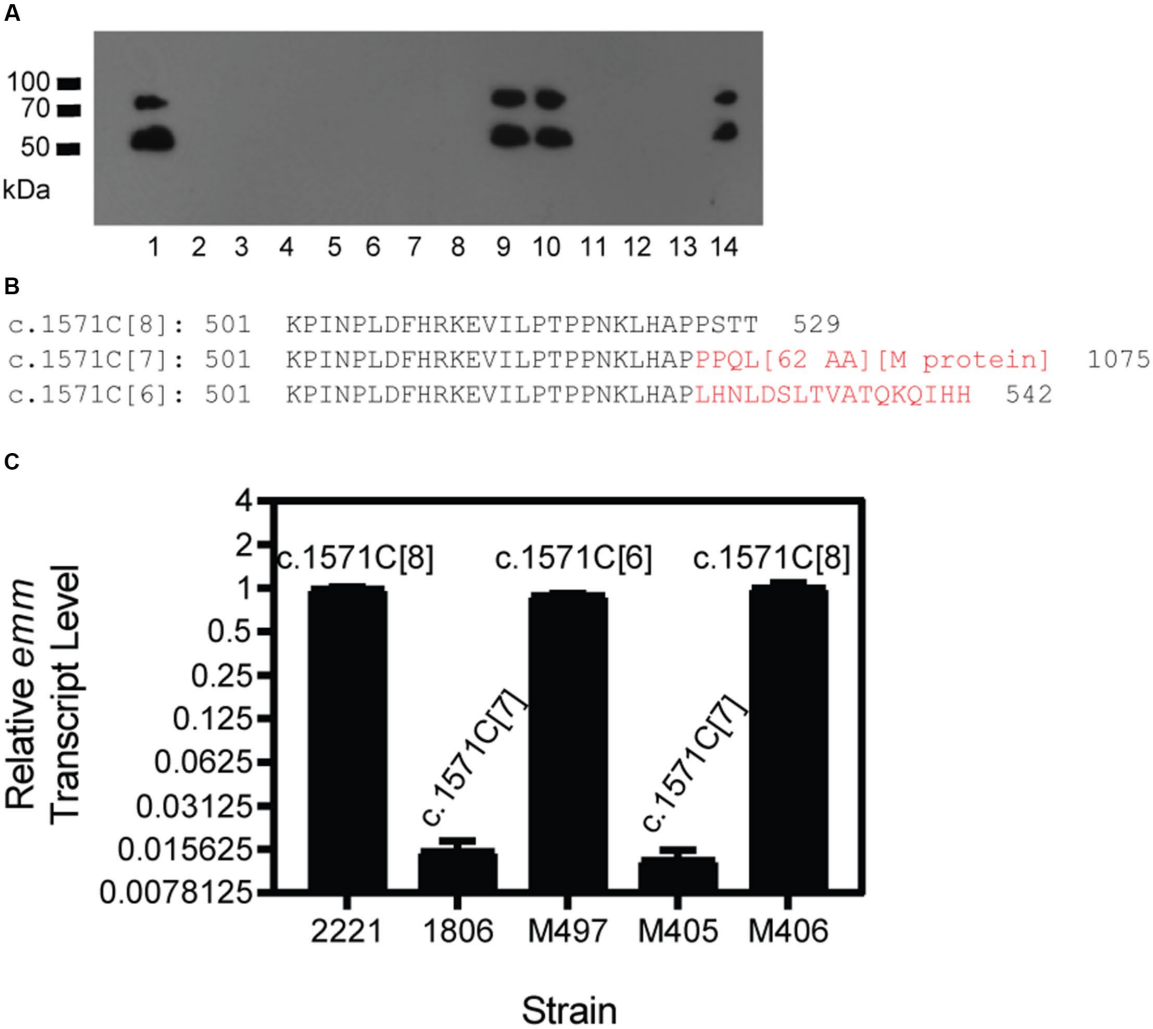
Selection of M^+^ GAS isolates from M^−^ GAS variants in infection sites of mice. Mice were inoculated subcutaneously with M^−^ c.1574C[7] variants of MGAS2221 or MGAS2221 Δ*sagA*, and GAS isolates from the infection site of the skin were tested for M protein production by Western blotting analyses. **(A)** Anti-M protein western blot of GAS isolates. Lanes: 1, MGAS2221; 2–14, isolates from skin infection site of M^−^ MGAS2221 ΔsagA. **(B)** The C-terminus Amino sequences starting from residue 501 for wild-type M1 Mga (c.1574C[8]) and its c.1574C[7] and c.1574C[6] variants. The sequences in red are different from that of the wild-type Mga, and [62AA] and [M protein] for c.1574C[7] variant represent amino acid sequence of the 66 amino acid residues encoded by the sequence between *mga* and *emm* and the sequence of the M protein, respectively. The numbers 529, 1,075 and 542 are the total number of amino acid residues in wild-type Mga and its c.1574C[7] and c.1574C[6] variants, respectively. **(C)** Relative emm mRNA levels of MGAS2221 (control), M^−^ GAS1806, and M^+^ M497 and M406 and M^−^ M405 isolated from GAS1806 infection site in the skin of mice. The polymorphism of the *mga* polycytidine tract is indicated.

A question of interest is whether the conversion of c.1571C[8] *mga* to c.1571C[7] *mga* is reversible. Since the M protein contributes to protection of GAS against the host innate immune response, *in vivo* growth would provide an environment to allow the conversion of c.1571C[7] *mga* to c.1571C[8] *mga*. To test this idea, two mice were subcutaneously infected with GAS1285, an M^−^ MGAS2221 Δ*sagA* mutant with the c.1571C[7] *mga* variant. GAS isolates were recovered from skin infection sites 4 days after inoculation, and 13 GAS colonies randomly chosen from each of mouse 1 and 2 for detecting M protein production by western blotting analysis. Three and four isolates among the analyzed isolates from mouse 1 (Figure 3A) and mouse 2 (western blot not shown) restored M protein production. DNA sequencing found that all 7 M^+^ isolates had c.1571C[8] *mga* (Table 3) whereas one M^−^ isolate from the infection was sequenced and still had the c.1571C[7] *mga* variant.

**Table 3:** Summary of selection of M^+^ isolates from skin infection site of mice with M^−^ GAS variants.

Mouse	Infection strain	Number of M^+^ isolates/total tested isolates from mouse	No. of M^+^ isolates with c.1571C[8] *mga*	No. of M^+^ isolates with c.1571C[6] *mga*
1	^a^GAS1285	3/13	3	0
2	GAS1285	4/13	4	0
3	^b^GAS1806	4/8	3	1
4	GAS1806	1/8	1	0
5	GAS1806	2/8	0	2
6	GAS1806	0/10		
7	GAS1806	0/10		

a GAS1285 was a M^-^ variant of MGAS2221ΔsagA with c.1574C[7] *mga* variant.

b GAS1806 was a M^-^ variant of MGAS2221 with c.1574C[7] *mga* variant.

We also tested GAS1806, an M^−^ c.1571C[7] variant of MGAS2221, in subcutaneous infection of 5 mice that are numbered from 3 to 7 in Table 3. Four of 20 isolates from mouse 3, 1 of 8 isolates from mouse 4, and 2 of 8 isolates from mouse 5 restored M protein production whereas 10 isolates from mouse 6 or mouse 7 did not have detectable M protein production. Three of the 4 M^+^ isolates from mouse 3 restored the c.1571C[8] tract of *mga*, and the other M^+^ revertant had an additional C deletion in the c.1571C[7] tract, resulting in 6 C at the polycytidine tract of *mga* that is designated as c.1571C[6]. The M^+^ isolate in mouse 4 restored the c.1571C[8] tract of *mga* whereas both M^+^ isolates in mouse 5 had the c.1571C[6] *mga* variant (Table 3). The additional 1C deletion of the c.1571C[7] tract of *mga* in the M^−^ variant shifted the reading frame, converting the Mga-62 AA-M1 protein fusion protein into an Mga variant of 542 aa residues, which was just 13 amino acid residues longer than the wild-type Mga protein of M1 GAS (Figure 3B). This c.1571C[6] *mga* variant apparently restored Mga function and thus the M protein production.

To test whether the c.1571C[7] and c.1571C[6] *mga* variants lost and regained function, *emm* mRNA levels were measured by real-time RT PCR for MGAS2221 (wt control), GAS1806 (c.1571C[7] *mga* variant of MGAS2221), and isolates M497, M405, and M406 from mouse infection with GAS1806. M497, M405, and M406 had c.1571C[6] (M497), c.1571C[7], and c.1571C[8] *mga* variants. As shown in Figure 3C, GAS1806 and M405 lost *emm* transcription by 98% whereas M497 and M406 restored *emm* transcription to 90 and 100% of that in MGAS2221. Thus, the c.1571C[7] *mga* variant is nonfunctional and can be reversed into functional c.1571C[8] *mga* by gaining 1\u00B0C or converted into functional c.1571C[6] *mga* variant by losing another C during mouse infection. The results indicate that the polycytidine tract at the 3′ end of the *mga* gene in M1 GAS can switch between c.1571C[8] or c.1574C[6] and c.1574C[7] to turn on and off M protein production, respectively.

### Polymorphisms of the polycytidine tract of *mga*-1 in sequenced GAS strains in genome databases

**Figure 4:**
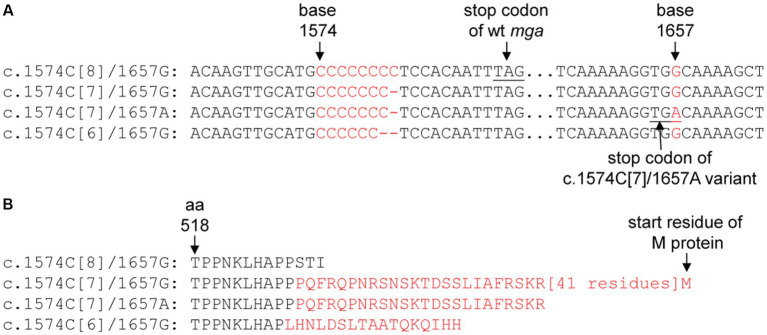
Polymorphism at the c.1574C[8] tract and base 1,657 of mga of sequenced M12 GAS genomes. **(A)** For different polymorphisms of the c.1574C[8] and base 1,657 in M12 *mga* genes. **(B)** The difference in amino acid sequence of Mga caused by the polymorphisms of mga in panel A. The sequences in red are those that are different from the wild-type Mga.

If the polycytidine tract-based switch of *mga* for phase variation in M protein production functions during infection, there should be polymorphism at the polycytidine tract of the *mga* gene in clinical isolates. M12 GAS frequently shows M protein phase variation *in vitro* (Simpson and Cleary, 1987). So we first examined the *mga* sequences of M12 GAS genomes in the NCBI genome database. The *mga* gene and protein sequences of 106 available M12 GAS genomes were aligned. As shown in Figure 4A, there are four different types of polymorphisms related to the number of the C repeats starting at base 1574 and polymorphism at base 1657: (1) functional c.1574C[8] *mga* and base G at position 1657 (1657G) (wt), (2) nonfunctional c.1574C[7] *mga* and 1657G (Variant 1), (3) functional c.1574C[6] *mga* and 1657G (Variant 2), and (4) functional *mga* variant with c.1574C[7] and 1657A (Variant 3). Wild-type Mga of M12 GAS has 530 amino acid residues. The nonfunctional c.1574C[7]*/*1657G Variant 1 encodes a nonfunctional Mga-M protein fusion protein with a 62-aa bridge encoded by the intergenic sequence between the *mga* and *emm* genes, the functional c.1574C[6]/1657G variant has 543 amino acid residues and is similar with the c.1571C[6] *mga* variants of MGAS2221 that were from the c.1574C[7] *mga* variant of MGAS2221 in mouse infection; and the G-to-A nonsense mutation at base 1657 of the c.1574C[7] *mga* variant converts Variant 1 into c.1574C[7]/1657A Mga variant that is 21 aa-residue longer than the wt Mga protein (Figure 4B) (Table 4). Among 106 M12 genomes in the NCBI genome database, there are 25 *wt mga*; 3 Variant 1, 38 Variant 2; and 40 Variant 3. These data are summarized in Table 4, and the *mga* polymorphisms at the polycytidine tract and base 1657 of 106 M12 genomes are listed in Supplementary Table S2. It is interesting that two of the three strains with nonfunctional c.1574C[7]/1657G *mga* variant 1, ATCC 11434 (Tanaka et al., 2020) and MGAS2096 (Sarkar et al., 2018), were isolated from patients with acute poststreptococcal glomerulonephritis, and the infection nature of the third strain, NCTC8300, is not known. The data indicate that the length polymorphism of Mga is caused through arising the nonfunctional c.1574C[7]/1657G *mga* variant and restoring the function of the nonfunctional c.1574C[7]/1657G *mga* variant by the additional C deletion at the c.1574C[7] tract or the G-to-A mutation at base 1657. These *mga* polymorphisms among clinical M12 isolates also indicate that M12 GAS undergoes phase variation in M protein production through the slipped-strand mispairing of the *mga* polycytidine tract.

**Table 4:** Polymorphism at the polycytidine tract and base 1,657 and Functionality of M1 and M12 *mga* among GAS genomes in the GenBank database.

M protein type genotype			M1	M12
Polymorphism of *mga* at the polycytidine and base 1,654 in M1 and base 1,657 in M12	^a^c.1571C[8]/1654G or c.1574C[8]/1657G	Frequency	150/158	25/106
		AA No. of Mga	529	530
		Functionality	Yes	Yes
	c.1571C[7]/1654G or c.1574C[7]/1657G	Frequency	3/158	3/106
		AA No. of MGA	1,075	1,157
		Functionality	No	No
	c.1571C[6]/1654G or c.1574C[6]/1657G	Frequency	5/158	38/106
		AA No. of Mga	542	543
		Functionality	Yes	Yes
	c.1571C[7]/1654A or c.1574C[7]/1657A	Frequency	0/158	40/106
		AA No of Mga		543
		Functionality		Yes

a The polycytidine tract with polymorphism starts at base 1,571 and 1,574 in M1 and M12 *mga*, respectively.

Among 158 M1 GAS strains (Supplementary Table S3), 5 M1 GAS strains, FDAARGOS, CCUG-4207, CCUG-4207-W1, NCTC8198, and AP1, had the functional c.1571C[6] *mga* variant, and three M1 strains, CCUG-47803, SPY8157, and GA41345, had the nonfunctional c.1571C[7] *mga* variant (Table 4 and Supplementary Table S3). Other M1 strains in the NCBI genome database have the wt c.1571C[8] *mga*. The frequency of the c.1571C[6] *mga* variant among the sequenced M1 GAS isolates, 5/158, is less than that among the sequenced M12 GAS isolates, 38/106 (Table 4). Four of 6 M14 GAS strains in the GenBank database have the wt c.1574C[8] *mga* encoding Mga with 530 amino acid residues, and two other strains have the c.1574C[6] *mga* variant for Mga with 543 amino acid residues (Table 1). One of two M23 strain has the c.1574C[6] *mga* variant for Mga with 543 amino acid residue, and another has 1C addition at the c.1574C[8] tract and 1C deletion at the c.1555C[6] tract, leading to c.1555C[5]/c.1573C[9] *mga* variant that encode Mga with 530 amino acid residues (Table 1). The c.1574C[6] *mga* variant in clinical M1, M14, and M23 GAS isolates indicates that the nonfunctional c1574C[7] *mga* variants of M1, M14, and M23 GAS arise during infection.

M3 GAS strains in the GenBank database have four polymorphisms of M3 *mga* that are related to the polycytidine tract near the 3′ end of *mga* (Supplementary Table S4). 106 strains have the wt c.1592C[8] *mga*; 5 strains have a missense mutation at base 1599 of the c.1592C[8] tract from C to T or c.1598C > T; 1 strain has an addition of 1C to the tract to result in c.1592C[9] *mga* variant; and two strains, A842 and A843, had 1C deletion at the c.1592C[8] tract and 1C addition at the c.1573C[6] tract to result in c.1592C[7]/c.1573[7] *mga* variant. It is unlikely that the two events in strains A842 and A843 occurred at the same time. These polymorphisms of M3 *mga* indicates the slipped-strand mispairing within the c.1592C[8] tract of M3 *mga* also occurs in hosts.

### Lack of polymorphism in Mga length for serotypes of GAS that do not have the *mga* polycytidine tract

The M49 *mga* gene belongs to the *mga*-2 cluster that does not have the polycytidine tract. Except for a three amino acid residue insertion near the C terminus of Mga in M49 strain K36294, all the Mga sequences of the other 29 M49 genomes do not show length polymorphism. The Mga sequences of the other *mga*-2, such as M89 *mga*, do not have the polycytidine tract, which is shown in the sequence alignment of *mga* of M1 strain MGAS5005 and M89 strain KUN-0012590 (Murase et al., 2020) (Supplementary Figure S2). M89 strains do not have polymorphism in the length of the Mga protein. These results indicate that the polycytidine tract of *mga*-1, but not *mga*-2, causes polymorphism in the length of Mga proteins.

## Discussion

**Figure 5:**
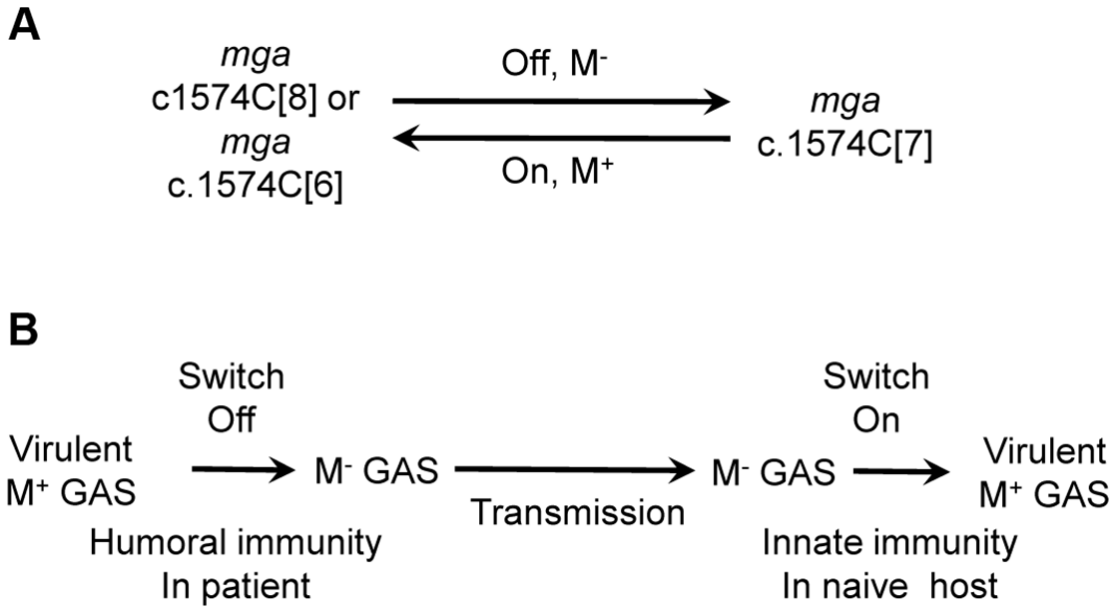
The cytidine repeat of *mga* as a switch for M protein production and the role of the switch in the circulation among human hosts. **(A)** Slipped-strand mispairing of the polycytidine tract of mga functions as a switch for M protein production. The c.1574C[8] and c.1574C[6] repeats of *mga* lead to functional Mga, which turns on M protein expression whereas c.1574C[7] leads to nonfunctional Mga, which turns off M protein expression. **(B)** Proposed role of the switch of M protein production in GAS circulation among human hosts. It is proposed that humoral immunity in patients turns off the switch, resulting in M^-^ variants that are transmitted to naïve host where innate immunity turns on the switch to produce M protein.

One of our findings is that the slipped-strand mispairing within the c.1571C[8] tract of the *mga* gene of M1 GAS mediates the phase variation of the M protein. *In vitro* genetic manipulation or culturing of M1 GAS strains frequently led to variants with the loss of M protein production. The majority of M^-^ variants had a single C base deletion in the c.1574C[8] tract of *mga*, leading to an open reading frame shift that combine the *mga* and downstream *emm*1 gene to code for a predicted Mga-M protein fusion. Complementation in trans of the c.1571C[7] *mga* variant with an *mga*-containing plasmid restored the production of the M protein. Isolates with restored M protein production were recovered at skin infection sites in mice with the c.1571C[7] *mga* variant, and the majority of these M^+^ revertants restored the c1571C[8] tract of *mga*. In addition, some M^+^ revertants lost another C, leading to a c.1571C[6] *mga* variant that codes for a Mga with additional 13 amino acid residues at the C-terminus compared with the wt Mga. Transcriptional data of *emm* indicates that the c.1571C[7] *mga* variant is nonfunctional whereas the c.1571C[6] *mga* variant is functional as an activator of *emm* transcription. In addition to arising of the c.1571C[7] *mga* variant, M protein production could be lost by the Mga T98P and T289P missense mutations, A-to-G mutation in the-10 box of the *emm* promoter, and unknown mutation(s) that affects post-transcriptional translation. The M protein of M12 GAS has been shown to have phase variation *in vitro* (Simpson and Cleary, 1987). Approximately 50-base pair deletions within or adjacent to the M protein coding sequence had been detected in two M^-^ variants but the detail of the deletions and whether the deletions were responsible for the loss of the M protein production were not known (Spanier et al., 1984). Our findings reveal that the slipped-strand mispairing within the c.1574C[8] tract in the *mga* gene of cluster 1 is one mechanism to reversibly turn the M protein production on and off (Figure 5A).

The M protein and several other virulence factors regulated by Mga are among genes that have the highest expressions according to transcriptional profiling with RNAseq analysis (Horstmann et al., 2018). The M protein, ScpA, and SclA are not essential for M1 GAS viability since their genes can be deleted (Li et al., 2013; Liu et al., 2015; Supplementary Table S1), and *emm* deletion mutant can survive in skin infection site of mice for several days (Liu et al., 2015). One possible reason for the arising of M^-^ variants in serial passage in THY is a metabolic burden driven evolutionary adaptation. In this case, the production of M protein constitutes a substantial anabolic burden that reduces fitness relative to strains that do not produce the M protein, and M^−^ mutants likely overgrow M protein-producing strains. We will examine this possibility in future. Loss of M protein production in construction of GAS gene deletion in our method using THY for passing significantly caused problems in our effort to generate gene deletion mutants without loss of M protein production in our previous studies. Fortunately, passing GAS with the first crossover on THY agar prevented the arising of M^-^ variants (Zhou et al., 2013; Supplementary Table S1). These results suggest that M^+^ GAS bacteria under limited nutrient condition may have defects in the envelope structure that compromise GAS survival in liquid. M^-^ variants may devote more nutrient to the synthesis of the cell wall to survive better in liquid under limited nutrient conditions. Passing on THY agar is an important improvement in the two step gene deletion approach to generate gene deletion mutants without loss of M protein production. Allelic exchange approach may have higher chance to obtain mutants with normal M protein production but introduces antibiotic selection marker. The *sagA* gene was shown to downregulate *emm* (Li et al., 2019). The *sda*1 gene was proposed to be selection pressure for *covRS* mutants (Walker et al., 2007). However, these results could not repeated, and loss of M protein production might be responsible for these observations (Zhou et al., 2013; Liu et al., 2015). No matter what approach is used to generate gene deletion mutants of GAS with *mga*-1, it is important to check *emm* transcription and M protein production of gene deletion mutants.

The M protein is required for resistance to phagocytosis by neutrophils (Perez-Casal et al., 1992). The M protein is important if not essential for growth *in vivo* in order to protect against the immune response, evolution has landed upon a reversible switch, so that when a mutant that has lost M protein production is back in an *in vivo* environment a portion of them will revert to producing the M protein helping to ensure the survival of the species. This explains why lack of M production enhances fitness *ex vivo*, but M protein production enhances fitness *in vivo*.

The polymorphism in the length of Mga among clinical GAS isolates indicates that the M protein phase variation through the slipped-strand mispairing within the c.1574C[8] tract occurs *in vivo*. Several clinical isolates of M1 and M12 GAS had the nonfunctional c.1574C[7] *mga* variant. It is possible that the c1574C[7] variant in these isolates might be acquired due to *in vitro* processing. However, the presence of the functional c.1574C[6] and c.1574C[7]/1657A *mga* variants indicate that the c.1574C[7] variant occurs *in vivo*. It is interesting that the 1657A allele is absent in the c.1574C[8] and c.1574C[6] *mga* variants. 37.7% of 106 M12 genomes in the GenBank database contain the c.1574C[7]/1657A *mga* variant. The majority of M12 strains with this *mga* variant should transcribe *emm* and produce M protein. Thus, it is reasonable to assume that the c.1574C[7]/1657A *mga* variant of M12 is functional. If this is true, the c.1657G > A nonsense mutation is somehow selected for functional *mga* variants from the nonfunctional c.1574C[7] *mga* variant. The M protein is required for resistance to phagocytosis, which is likely one of selection pressures for functional Mga from c.1574C[7] *mga* variant. Apparently, there is no selection pressure to select the c.1574G > A mutation in c.1574C[8] and c.1574C[6] because the c.1574G > A mutation does not alter the functionality of the functional *mga* variants.

The C-terminal region of Mga is important for oligomerization of Mga and transcriptional activation (Hondorp et al., 2012). Fusion of an extra 546 amino acid residues to the C-terminus of Mga in the c.1571C[7] *mga* variant of M1 and M12 GAS is expected to interfere with Mga oligomerization and transcriptional activation. The M12 c.1574C[7]/1657A *mga* variant encodes the Mga variant of 551 amino acid residues, 20 amino acid residues longer than the wt M12 *mga*, suggesting that addition of short peptides to the C-terminus of Mga does not affect the function of Mga dramatically.

It is not known why the nonfunctional c.1574C[7] *mga* variant arises and functional c.1574C[7]/1657A and c.1574C[6] *mga* variants are selected in host. We speculate that the acquired immunity may play a role in this process. Anti-M protein antibodies enhance GAS killing by neutrophils and may confer an advantage to M^-^ variants. We do not have experimental evidence for this possibility. It is also possible that the c.1574C[7] *mga* variant arises in a nutrient-limited niche in the host. If the humoral immunity indeed plays a role for the c.1574C[7] *mga* variant, the selection of the functional c.1574C[8], c.1574C[6] and c.1574C[7]/1657A *mga* variants from c.1574C[7] *mga* variant would not occur in the same host in which the c.1574C[7] variant is. It is more likely that the c.1574C[7] *mga* variant is transmitted into naïve hosts where the functional *mga* variants arise in response to the innate immune responses like in the mouse infection. It is proposed that the reversible switch of the M protein phase variation through the slipped-strand mispairing within the c.1574C[8] tract of the *mga* gene plays a role in the continued circulation of GAS among human hosts (Figure 5B). In this proposal, GAS with the c.1574C[8] *mga* is transmitted from patients to naïve hosts to cause acute infections, and the humoral immunity then selects the c.1574C[7] *mga* variant; and the c.1574C[7] *mga* variant is transmitted to new naïve hosts and converted into c.1574C[8] to cause infection. It would be interesting to test this proposal in future work.

Slipped-strand mispairing within a repetitive sequence is commonly used for adaption of bacteria to particular environment or niche. For Group A Streptococcus, slipped-strand mispairing in AACAA repeats in coding region controls production of streptococcal collagen-like protein B (Lukomski et al., 2001; Rasmussen and Björck, 2001). The well-characterized hypervirulent M1T1 GAS strain MGAS5005 had a T deletion in the 7 T tract starting at base 77 of the *covS* gene (Li et al., 2013), and the T deletion variants of *covS* were found at GAS infection site of mice (Engleberg et al., 2001). Slipped-strand mispairing within repetitive sequences appears to be a common mechanism for selecting variants that have advantage for survival under particular stress. Our findings represent an unusual mechanism that the slipped-strand mispairing in *mga* occurs near the end of the *mga* gene. This arrangement apparently can interfere with the role of the C-terminus of Mga in oligomerization and transcriptional activation but at the same time confers reversibility of the process.

## Conclusion

*In vitro* manipulation of GAS frequently leads to the loss of M protein production. Deletion of base C within the polycytidine tract c.1571C[8] of *mga* result in the nonfunctional c.1571C[7] *mga* variant, turning off M protein production. M^+^ isolates were obtained from infection sites with M^−^ GAS variants in mice and either restored the wild-type c.1574C[8] tract of *mga* or had a functional c.1574C[6] *mga* variant due to an additional C deletion at the c.1574C[7] tract. It is concluded that slipped-strand mispairing within the c.1571C[8] tract of M1 *mga* functions as a switch for the M protein phase variation. The nonfunctional c.1574C[7] and functional c.1574C[6] variants are present in M1, M12, M14, and M23 strains in the GenBank databases, and a G-to-A nonsense mutation at base 1,657 of c.1574C[7] *mga* leads to a functional c.1574C[7]/1657A *mga* variant and is common in clinical M12 isolates. These polymorphisms of the polycytidine tract and base 1,657 of *mga* indicate that M protein phase variation through the slipped-strand mispairing within the c.1574C[8] tract occurs *in vivo*.

## Data availability statement

Publicly available datasets were analyzed in this study. This data can be found at: Genomes of Group A Streptococcus in NCBI GenBank database were used in analyses.

## Ethics statement

The animal study was reviewed and approved by Montana State University, Bozeman IACUC.

## Author contributions

Experiments were designed by BL and performed by TH, ML, and BL. Bioinformatic analyses were done by YB. The manuscript was written by BL and TH. All authors contributed to the article and approved the submitted version.

## Funding

This work was supported in part by grant AI153755 from the National Institutes of Health and the Montana State Agricultural Experimental Station.

## Conflict of interest

The authors declare that the research was performed in the absence of any commercial or financial relationships that could be construed as a potential conflict of interest.

## Publisher’s note

All claims expressed in this article are solely those of the authors and do not necessarily represent those of their affiliated organizations, or those of the publisher, the editors and the reviewers. Any product that may be evaluated in this article, or claim that may be made by its manufacturer, is not guaranteed or endorsed by the publisher.

## Footnotes

^1^ https://ftp.ncbi.nlm.nih.gov/genomes/genbank/bacteria/Streptococcus_pyogenes

^2^ https://ftp.cdc.gov/pub/infectious_diseases/biotech
