# Supplementary material for: Slipped-strand mispairing within a polycytidine tract in transcriptional regulator mga leads to M protein phase variation and Mga length polymorphism in Group A Streptococcus
Source: Front Microbiol. 2023 Jun 26;14:1212149. doi: 10.3389/fmicb.2023.1212149 (PMC10330708; doi:10.3389/fmicb.2023.1212149)
Supplement: Supplementary file 1 [file Data_Sheet_1.docx]

Supplementary Material for

Slipped-strand mispairing within a polycytidine tract in transcriptional activator *mga* leads to M protein phase variation and Mga length polymorphism in Group A *Streptococcus*

Benfang Lei^1^*, Tracey S. Hanks^1^, Yunjuan Bao^2^, Mengyao Liu^1^

^1^Department of Microbiology and Cell Biology, Montana State University, Bozeman, MT, USA

^2^State Key Laboratory of Biocatalysis and Enzyme Engineering, School of Life Sciences, Hubei University, Wuhan, Hubei Province, China

* Address Correspondence to Benfang Lei: [blei@montana.edu](mailto:blei@montana.edu)

Table of Contents

Supplemental Table 1………………………………………………………………………………..3

Supplemental Table 2………………………………………………………………………………..9

Supplemental Table 3……………………………………………………………………………….11

Supplemental Table 4………………………………………………………………………………..13

Supplemental Figure 1………………………………………………………………………...…….14

Supplemental Figure 2……………………………………………………………………………….15

Supplemental Table 1. GAS gene deletion mutants: M protein production, *emm* mRNA, polymorphism of *mga* and other mutations

| Strain No. | Strains | Passage medium | M protein production | relative mRNA | *mga* polymorphism | ^f^Reference |
| --- | --- | --- | --- | --- | --- | --- |
| MGAS2221 | wt |  | + | 1 | ^a^c.1571C[8] |  |
| GAS874 | MGAS2221Δsse | THY | - | 0.055 | ^c^c.292A>C |  |
| GAS875 | GAD2221Δsse | THY | + | 0.95 | c.1571C[8] | Li et al. 2013 |
| GAS1099 | MGAS2221 ΔscpA | THY | - | 0.021 | ^d^-74A>G of *emm* |  |
| GAS1568 | MGAS2221ΔscpA | THY | + | 1.2 | c.1571C[8] | Li et al. 2013 |
| GAS1213 | MGAS2221 ∆spyCEP | THY | - | 0.02 | ^b^c.1571C[7] |  |
| GAS1214 | MGAS2221ΔspyCEP | THY | - | 0.022 | c.1571C[7] |  |
| GAS1548 | MGAS2221ΔspyCEP | THY | + | 0.98 | c.1571C[8] | Li et al. 2013 |
| GAS1208 | MGAS2221ΔspyCEP | THY | + | 1.01 | c.1571C[8] |  |
| GAS1238 | MGAS2221ΔcovS | THY | + |  | c.1571C[8] | Li et al., 2013 |
| GAS1128 | MGAS2221ΔsagA | THY | - | 0.01 | c.1571C[7] | Zhou et al., 2013 |
| GAS1255 | MGAS2221ΔsagB | THY | - | 0.011 | c.1571C[7] | Zhou et al., 2013 |
| GAS1256 | MGAS2221ΔsagB | THY | + | 1.2 | c.1571C[8] | Zhou et al., 2013 |
| GAS1257 | MGAS2221ΔsagB | THY | + | 1.1 | c.1571C[8] | Zhou et al., 2013 |
| GAS1285 | MGAS2221ΔsagA | THY | - | 0.013 | c.1571C[7] | Zhou et al., 2013 |
| GAS1286 | MGAS2221ΔsagA | THY | - | 0.012 | c.1571C[7] | Zhou et al., 2013 |
| GAS1546 | MGAS2221ΔsagA | THY agar | + | 0.92 | c.1571C[8] | Zhou et al., 2013 |
| GAS1547 | MGAS2221ΔsagA | THY agar | + | 0.88 | c.1571C[8] | Zhou et al., 2013 |
| Supplemental Table 1. Continued | | | | | | |
| Strain No. | Strains | Passage medium | M protein production | relative mRNA | mga polymorphism | Reference |
| GAS1473 | MGAS2221Δscl | THY | - |  | c.1571C[7] |  |
| GAS1474 | MGAS2221Δscl | THY | - |  | c.1571C[7] |  |
| GAS1475 | MGAS2221Δscl | THY | - |  | c.1571C[7] |  |
| GAS1476 | MGAS2221Δscl | THY | - |  | c.1571C[7] |  |
| GAS1478 | MGAS2221Δscl | THY | - |  | c.1571C[7] |  |
| GAS1551 | MGAS2221Δscl | THY | + |  |  |  |
| GAS1582 | MGAS2221ΔScl | THY agar | + |  |  |  |
| GAS1583 | MGAS2221Δscl | THY agar | + |  |  |  |
| GAS1498 | MGAS2221Δspy1118 | THY | - |  | c.1571C[7] |  |
| GAS1499 | MGAS2221Δspy1118 | THY | - |  | c.1571C[8] |  |
| GAS1500 | MGAS2221Δspy1118 | THY | - |  | c.1571C[7] |  |
| GAS1504 | MGAS2221Δspy1118 | THY | + |  | c.1571C[8] |  |
| GAS1505 | MGAS2221∆spy1118 | THY | - |  | c.1571C[7] |  |
| GAS1506 | MGAS2221∆spy1118 | THY | - |  | c.1571C[7] |  |
| GAS1507 | MGAS2221∆spy1118 | THY | - |  | c.1571C[7] |  |
| GAS1508 | MGAS2221∆spy1118 | THY | - |  | c.1571C[7] |  |
| GAS1603 | MGAS2221Δsda1spd3 | THY agar | - |  | c.1571C[8] | Liu et al., 2015 |
| GAS1604 | MGAS2221Δsda1spd3 | THY agar | + |  | c.1571C[8] | Liu et al., 2015 |
| GAS1605 | MGAS2221Δsda1spd3 | THY agar | + |  |  |  |
| GAS1573 | MGSA2221Δsda1 | THY | + |  |  | Liu et al., 2015 |
| Supplemental Table 1. Continue | | | | | | |
| Strain No. | Strains | Passage medium | M protein production | relative mRNA | mga polymorphism | Reference |
| GAS1682 | MGAS2221ΔhasA | THY agar | + |  | c.1571C[8] | Liu et al., 2015 |
| GAS1683 | MGAS2221ΔhasA | THY agar | + |  |  |  |
| GAS1684 | MGAS2221ΔhasA | THY agar | + |  |  |  |
| GAS1685 | MGAS2221ΔhasA | THY agar | + |  |  |  |
| GAS1686 | MGAS2221ΔhasA | THY agar | + |  |  |  |
| GAS1687 | MGAS2221ΔhasA | THY agar | + |  |  |  |
| GAS1688 | MGAS2221ΔhasA | THY agar | + |  |  |  |
| GAS1694 | MGAS2221ΔhasA | THY agar | + |  |  |  |
| GAS1695 | MGAS2221ΔhasA | THY agar | + |  |  |  |
| GAS1696 | MGAS2221ΔhasA | THY agar | + |  |  |  |
| GAS1697 | MGAS2221ΔhasA | THY agar | + |  |  |  |
| GAS1698 | MGAS2221ΔhasA | THY agar | + |  |  |  |
| MGAS5005 | wt |  | + | 1 | c.1571C[8] |  |
| GAS691 | MGAS5005Δsse | THY | + | 1.2 | c.1571C[8] | Zhu et al., 2009 |
| GAS895 | MGAS5005ΔcovS | THY | + |  | c.1571C[8] | Li et al., 2013 |
| GAS 920 | MGAS5005^wt^covS | THY | + |  | c.1571C[8] | Li et all., 2013 |
| GAS 921 | MGAS5005^wt^covS | THY | - |  | c.1571C[7] |  |
| GAS 932 | MGAS5005ΔsagA | THY | + |  | c.1571C[8] | Zhou et al., 2013 |
| GAS 1042 | MGAS5005ΔspyCEPΔscpA | THY | + | 1.1 | c.1571C[8] | Li et al., 2013 |
| Supplemental Table 1. Continued | | | | | | |
| Strain No. | Strains | Passage medium | M protein production | relative mRNA | mga polymorphism | Reference |
| GAS 1067 | MGAS5005ΔspyCEPΔscpAΔSSE | THY | - | 0.015 | c.1571C[7] |  |
| GAS 1068 | MGAS5005ΔspyCEPΔscpAΔSSE | THY | + |  | c.1571C[7] |  |
| GAS 1069 | MGAS5005ΔspyCEPΔscpAΔSSE | THY | + |  | c.1571C[8] | Li et al., 2013 |
| GAS 1081 | MAS5005ΔsagAΔscpA Δsse | THY | - |  | c.1571C[8] |  |
| GAS 1092 | MGAS5005ΔspyCEPΔspysse | THY | + |  |  | Li et al., 2013 |
| GAS 1093 | MGAS5005ΔspyCEPΔspysse | THY | + |  |  |  |
| GAS 1133 | MGAS5005Δsse | THY | - |  | c.1571C[7] |  |
| GAS 1134 | MGAS5005Δsse | THY | - |  | c.1571C[7] |  |
| GAS 1135 | MGAS5005Δsse | THY | - |  |  |  |
| GAS 1136 | MGAS5005Δsse | THY | - |  | c.1571C[7] |  |
| GAS 1137 | MGAS5005Δsse | THY | - |  | c.1571C[7] |  |
| GAS 1138 | MGAS5005Δsse | THY | - |  | c.1571C[7] |  |
| GAS 1146 | MGAS5005ΔscpAΔsse | THY | + |  | c.1571C[8] | Li et al., 2013 |
| GAS 1147 | MGAS5005ΔscpAΔsse | THY | - |  | c.1571C[7] |  |
| GAS 1148 | MGAS5005ΔscpAΔsse | THY | - |  | c.1571C[7] |  |
| GAS 1149 | MGAS5005ΔscpAΔsse | THY | + |  |  |  |
| GAS 1185 | MGAS5005^wt^covRSΔsagA | THY | - |  | c.1571C[8] |  |
| GAS 1560 | MGAS5005Δsse | THY agar | + | 1.3 |  | Liu et al., 2012 |
| GAS 1561 | MGAS5005Δsse | THY agar | + |  |  |  |
| GAS 1562 | MGAS5005Δsse | THY agar | + |  |  |  |
| Supplemental Table 1. Continued | | | | | | |
| Strain No. | Strains | Passage medium | M protein production | relative mRNA | mga polymorphism | Reference |
| GAS 1563 | MGAS5005Δsse | THY agar | + |  |  |  |
| GAS 1564 | MGAS5005Δsse | THY agar | + |  |  |  |
| GAS 1565 | MGAS5005Δsse | THY agar | + |  |  |  |
| GAS1566 | MGAS5005Δsse | THY agar | + |  |  |  |
| GAS1567 | MGAS5005Δsse | THY agar | + |  |  |  |
| GAS550 | MGAS5005Δspy1870 | THY | - |  | c.1571C[7] |  |
| 5448 | wt |  | + | 1 | c.1571C[8] | Liu et al., 2015 |
| GAS1778 | 5448Δsda1 | THY | - | 0.97 | c.1571C[8] | Liu et al., 2015 |
| GAS1781 | 5448Δsda1 | THY agar | + | 0.99 | c.1571C[8] | Liu et al., 2015 |
| SF370 | wt |  | + |  | c.1571C[8] |  |
| GAS827 | SF370Δcovs | THY | - |  | c.1571C[7] |  |
| GAS1458 | SF370Δscl | THY | - |  | c.1571C[7] |  |
| GAS1459 | SF370Δscl | THY | - |  | c.1571C[7] |  |
| GAS1514 | SF370Δscl | THY | - |  | c.1571C[7] |  |
| GAS1515 | SF370Δscl | THY | - |  | c.1571C[7] |  |
| GAS1516 | SF370Δscl | THY | - |  | c.1571C[7] |  |
| GAS1517 | SF370Δscl | THY | - |  | c.1571C[7] |  |
| GAS1518 | SF370Δscl | THY | - |  | c.1571C[7] |  |
| GAS1519 | SF370Δscl | THY | + |  | c.1571C[8] |  |
| Supplemental Table 1. Continued | | | | | | |
| Strain No. | Strains | Passage medium | M protein production | relative mRNA | mga polymorphism | Reference |
| MGAS315 | wt |  |  | 1 |  | Feng et al., 2017 |
| GAS990 | MGAS315ΔsagA | THY |  | 1.2 | ^e^c.1592C[8] | Feng et al., 2017 |
| GAS918 | MGAS315Δsse | THY |  | 0.9 | c.1592C[8] | Feng et al., 2017 |
| GAS1072 | MGAS315ΔsseΔsagA | THY |  | 1.1 | c.1592C[8] | Feng et al., 2017 |
| GAS1191 | MGAS315^wt^covS | THY |  | 0.01 | c.1592C[7] |  |
| GAS1717 | MGAS315ΔcovS | THY agar |  | 0.9 |  | Stetzner et al., 2015 |
| GAS1746 | MGAS315^wt^covS | THY agar |  | 0.85 | c.1592C[8] | Stetzner et al., 2015 |
| GAS1292 | MGAS315ΔropB | THY |  | 1.4 |  | Stetzner et al., 2015 |
| GAS1798 | MGAS315^wt^ropB | THY agar |  | 1.1 | c.1592C[8] | Stetzner et al., 2015 |
| GAS1028 | MGAS315ΔpyCEPΔsse | THY |  | 0.013 | c.1592C[7] |  |

^a^c.1571C[8] is the wild polycytidine tract starting at base 1571 of M1 *mga*.

^b^c.1571C[7] is the mga variant that has 1C deletion at the c.1571C[8] tract.

^c^Missense mutation at base 292 from A to C that led Mga T98P mutation.

^d^The A-to-G mutation occurred at base 74 upstream of the emm gene.

^e^c.1592C[8] is the polycytidine tract of M3 *mga* that is corresponding to the c.1571C[8] in M1 *mga*.

^f^The details of the references are listed in the references of the main text.

Supplemental Table 2. M12 strain list of polymorphisms at the polycytidine tract and base 1657

| c.1574C[8]/1657G | c.1574C[7]/1657G | c.1574C[7]/1657A | c.1574C[6]/1657A |
| --- | --- | --- | --- |
| HLJGAS12011 | MGAS2096 | 20154129-W1 | HKU383 |
| HKU360 | ATCC11434-W1 | BJCYGAS15 | 20154137-W1 |
| HKU388 | NCTC8300 | GAS06166 | NCTC5163 |
| HKU30 |  | GAS1441 | NCTC8332 |
| HKU306 |  | HKU16 | NCTC5164 |
| HKU397 |  | HKU22 | GASEMM0726 |
| NS3785 |  | NGAS096 | FDAARGOS-534-W1 |
| A1138 |  | NGAS145 | FDAARGOS-668-W1 |
| A1144 |  | NGAS447 | 20154031-W1 |
| NS488 |  | NGAS749 | 20154035-W1 |
| NS4518 |  | GASEMM1445 | 20154049-W1 |
| HKU165 |  | ABC020047993 | 970-SDYS |
| NCTC10085 |  | 20156447-W1 | Bra011 |
| GASEMM2115 |  | ABC020017280 | Bra051 |
| CCUG-25570 |  | GASEMM1434 | GAS09422 |
| GAS0617 |  | ABC020031898 | GAS09437 |
| GAS09398 |  | 20160140-W1 | GAS14243 |
| PHE-12749-W1 |  | 20156410-W1 | GAS146 |
| PHE-33064-W1 |  | GASEMM2898 | GASEMM2093 |
| PHE-33069-W1 |  | PHE-12750-W1 | GASEMM2100 |
| SP1336 |  | 20154129-W1 | GASEMM2260 |
| TJ11-001 |  | BJCYGAS15 | GASEMM2311 |
| A879 |  | GAS06166 | GASEMM0619 |
| MUMCMC661 |  | GAS1441 | GASEMM0274 |
| 20160981-W1 |  | HKU16 | GASEMM0568 |
|  |  | HKU22 | HKU364 |
|  |  | NGAS096 | MGAS9429 |
|  |  | NGAS145 | MUMCMC1953 |
|  |  | NGAS447 | PHE-25109-W1 |
| Supplemental Table 2. Continued. | | | |
| c.1574C[8]/1657G | c.1574C[7]/1657G | c.1574C[7]/1657A | c.1574C[6]/1657A |
|  |  | NGAS749 | PHE-32280-W1 |
|  |  | GASEMM1445 | PHE-45825-W1 |
|  |  | ABC020047993 | PHE-12021-W1 |
|  |  | 20156447-W1 | 20154148-W1 |
|  |  | ABC020017280 | 20154168-W1 |
|  |  | GASEMM1434 | 20154946-W1 |
|  |  | ABC020031898 | 20160945-W1 |
|  |  | 20160140-W1 | NGAS185 |
|  |  | 20156410-W1 | NGAS260 |
|  |  | GASEMM2898 |  |
|  |  | PHE-12750-W1 |  |

Supplemental Table 3. List of 158 *emm*1 strains that were analyzed for *mga* and Mga polymorphism

| Strain | Strain | Strain | Strain | Strain |
| --- | --- | --- | --- | --- |
| ^a^AP1 | SPY6025 | MUMCMC13 | Bra032 | ABC020046688 |
| ^a^FDAARGOS-774-W1 | SPY6018 | MGAS5005 | Bra019 | ABC020046986 |
| ^a^NCTC8198 | SPY6016 | M1-476 | Bra009 | ABC020047925 |
| ^a^CCUG4207-W1 | SPY6013 | Lacen1 | A20 | ABC020047955 |
| ^a^CCUG-4207 | SPY5448 | HKU488 | A1268 | ABC020047977 |
| ^b^CCUG-47803 | SPY2006 | HKU488 | 5448-W1 | ABC020048387 |
| ^b^SPY8157 | SP5-LAU | HKU487 | 5448 | ABC020048503 |
| ^b^GA41345 | SC08 | HKU486 | 20162629-W1 | ABC020049250 |
| 20154623-W1 | SC06 | HKU484 | 20161065-W1 | ABC020049545 |
| Lacen3 | SC05 | HKU474 | 20156414-W1 | ABC020050193 |
| NGAS056 | S119 | HKU464 | 20154821-W1 | ABC020051161 |
| NGAS202 | PHE-33033-W1 | HKU434 | 20154803-W1 | ABC020052291 |
| NGAS255 | PHE-12806-W1 | HKU419 | 20154557-W1 | ABC020052309 |
| NGAS413 | patientB | GAS12271 | 20154127-W1 | ABC020052313 |
| NGAS657 | NS696 | GAS12252 | ABC020005716 | ABC020052375 |
| NGAS336 | NS4697 | GAS12243 | ABC020005887 | ABC020052543 |
| Lacen2 | NGAS750 | GAS12233 | ABC020006345 | ABC020052553 |
| GASEMM1878 | NGAS623 | GAS06220 | ABC020006715 | ABC020052877 |
| GASEMM0832 | NGAS450 | GAS05134 | ABC020013256 | ABC020052898 |
| GASEMM0136 | NGAS425 | FDAARGOS-149 | ABC020014764 | ABC020052980 |
| 20162109-W1 | NGAS408 | emm1-W1 | ABC020015294 | ABC020053250 |
| 20154787-W1 | NGAS305 | emm1-S9-W1 | ABC020017774 | ABC020056020 |
| SF370 | NGAS297 | emm1-S8-W1 | ABC020021452 | ABC020056068 |
| 20162642-W1 | NGAS083 | emm1-S7-W1 | ABC020029711 | ABC020056181 |
| 20161447-W1 | NGAS078 | emm1-S5-W1 | ABC020030020 | ABC020056883 |
| ABC020056765 | NGAS076 | emm1-S18-W1 | ABC020032057 | ABC020056885 |
| 20154145-W1 | NGAS061 | emm1-S14-W1 | ABC020032535 | ABC020057288 |
| SPY5006C | NGAS035 | emm1-S11-W1 | ABC020038545 | ABC020057442 |
| SPY5006B | NCTC8370 | emm1-S10-W1 | ABC020041397 | ABC020059502 |
| SPY5006A | MUMCMC662 | emm1-NS7-W1 | ABC020044173 | ABC020061424 |
| PHE-30630-W1 | MUMCMC616 | emm1-NS6-W1 | ABC020046470 |  |
| GAS06216 | MUMCMC51 | Bra048 | Lacen4 |  |

^a^Strains that have the c.1571C[6] *mga* variant; ^b^strains that have the c.1571C[7] *mga* variant; and the other strains that have the wt c.1571C[8] *mga*.

Supplemental Table 4. List of 113 M3 GAS strains analyzed for for *mga* and Mga polymorphism

| Strain | Strain | Strain | Strain | Strain |
| --- | --- | --- | --- | --- |
| ^a^NS2403 | ABC020047619 | 20155031-W1 | NGAS100 | ABC020047959 |
| ^a^NS2299 | ABC020006103 | 20154799-W1 | ABC020060793 | ABC020047395 |
| ^a^NS2283 | 20154813-W1 | 20154166-W1 | ABC020057449 | ABC020047328 |
| ^a^A843 | GAS12171 | 20154133-W1 | ABC020057192 | ABC020047076 |
| ^a^A842 | STAB902 | NGAS650 | ABC020057168 | ABC020046589 |
| ^b^ABC020025676 | ABC020052519 | PHE-30587-W1 | ABC020056898 | ABC020044203 |
| ^c^ABC020044412 | ABC020017526 | PHE-45817-W1 | ABC020056891 | ABC020044193 |
| ^d^PHE-12088-W1 | ABC020014925 | PHE-12912-W1 | ABC020056064 | ABC020044188 |
| PHE-12833-W1 | SSI-1 | PHE-12907-W1 | ABC020056060 | ABC020044010 |
| PHE-12026-W1 | SPY6004 | NGAS104 | ABC020055975 | ABC020043540 |
| NGAS094 | SPY5626 | PHE-12888-W1 | ABC020055897 | ABC020038558 |
| PHE-18958-W1 | SPY2028 | PHE-45826-W1 | ABC020054955 | ABC020033020 |
| GASEMM2682 | PHE-12861-W1 | PHE-32261-W1 | ABC020053240 | ABC020032182 |
| GASEMM2370 | NGAS419 | PHE-39585-W1 | ABC020052558 | ABC020030063 |
| A873 | MGAS315 | PHE-27701-W1 | ABC020052497 | ABC020026799 |
| A856 | M3KCL | PHE-12908-W1 | ABC020052420 | ABC020026287 |
| SPY8003 | M3-b | PHE-12894-W1 | ABC020052023 | ABC020015292 |
| KS030 | GASEMM1189 | PHE-12890-W1 | ABC020051269 | ABC020014897 |
| GASEMM0963 | GAS1218 | PHE-12826-W1 | ABC020050818 | ABC020013551 |
| GASEMM2438 | GAS12172 | PHE-12805-W1 | ABC020049540 | ABC020006298 |
| FDAARGOS-514-W1 | GAS0327 | PHE-12062-W1 | ABC020048541 | ABC020005773 |
| PHE-26041-W1 | GAS0320 | NGAS426 | ABC020048395 | ABC020004984 |
| ABC020052378 | GAS0317 | NGAS266 |  |  |

^a^Strains has a C-to-T missense mutation, or c.1592C[7] and an T insertion at base 1599, resulting in no change in the length of Mga

^b^Strain has c.1592C[7] and an insertion of C at base 1573, which convert c.1573C[6] into c.1573C[7], resulting in no change in the length of Mga

^c^The strain has c.1592C[9] mga, an addition of C that leads to a Mga variant of 550 amino acid residues.

dThe other 106 strains have the wt c.1592C[8] mga that encodes Mga of 536 amino acid residues.


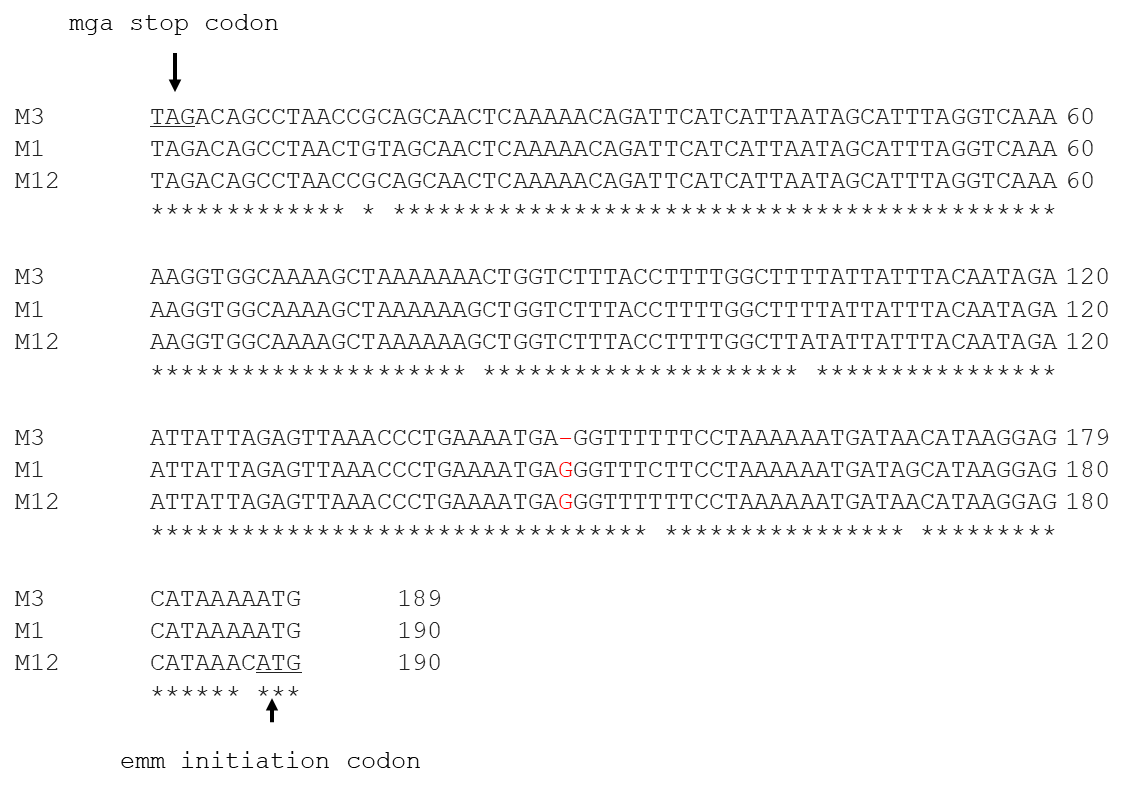


Supplemental Figure 1. Alignment of DNA sequences between the stop codon of the mga gene and the initiation codon of the emm gene from M3 (MGAS315), M1 (MGAS5005), and M12 (HKU360) strains. * represents identical base among the three sequences. G and – in red indicate the deletion of G in the M3 sequence.

M1 GCATTTGTAGCAGGTGTAGAAAAACTGATGCGTTCAGAGAAAGAACCTATCAGCACAGAG 1140

M89 GCCTTTGTGACAAGTGTCGAGAAGCTGATGCAGGCAGATAATGCTCAAGTTAGCAAAGAA 1098

** ***** ** **** ** ** ******* **** ** * * * **** ***

M1 TTGACTAACCAATTGATCTACGCCTTTTTCATCACTTGGGAAAATAGTTTCCTAAAAGTA 1200

M89 TTGATCAATCAGCTCACTTATTGTTTCTTTATTACCTGGGAAAATAGTTTCTTAAAAGTA 1158

**** ** ** * * ** ** ** ** ** *************** ********

M1 AATCAAAAAGATGAAAAAATTCGTCTTCTGGTGATTGAAAGAAGTTTTAACAGTGTTGGT 1260

M89 AATCAAAAAGATGAAAAAGTACGACTCCTAGTGATAGAGAGAAGTTACAATAGTGTCGGT 1218

****************** * ** ** ** ***** ** ******* ** ***** ***

M1 AATTTCCTAAAAAAGTACGTCGGAGAGTTTTTTAGCATCACAAACTTCAATGAGCTAGAT 1320

M89 AATTTTTTGAAAAAGTACATCGGTGAGTTTTTCAGCATTACTAATTTTGATGAGTTAGAT 1278

***** * ********* **** ******** ***** ** ** ** ***** *****

M1 GCTCTGACCATCGATCTAGAAGAGATTGAAAAACAGTATGATGTGATCGTGACAGATGTT 1380

M89 TGTTTGACAATTGATCTAGTAGAGATTGAAAAACAGTACGATGTTATCGTGACAGATGTT 1338

* **** ** ******* ****************** ***** ***************

M1 ATGGTAGGAAAAAGCGAAGAGCTAGAAATTTTCTTTTTCCACAAAATGATTCCAGAAGCG 1440

M89 ATGGTGGGTAAAAGCGAAGAGTTAGAGATTTTCTTTTTCTACAAGATGATCCCAGAAGCT 1398

***** ** ************ **** ************ **** ***** ********

M1 ATTATTGACAAGCTCAATGCGTTTTTAAACATCAGCTTTGCAGACAGCTTGCCACTAGAC 1500

M89 ATTATTGATAGATTAAATGAATTTTTGAACGTTAGTTTCACAGATAATAACGTTATGGTC 1458

******** * * **** ***** *** * ** ** **** * * * *

M1 AAACCCATCAACCCCTTGGACTTTCATCG---------CAAAGAG--GTTATCTTACCCA 1549

M89 AAACCCCTCGAAGCCCCTTCCTCTTCAAAATCTCATAGCGACAAAGAGGTACAAAAGCCA 1518

****** ** * ** ** * * * * * ** * ***

M1 CTC-CCCCCAACAAGTTGCATGCCCCCCCCTCCACAACTTAG 1590

M89 GAAAAGCCAGACAATTCAGTTAATCAAGCAACATCATCATAG 1560

** **** * * * * * ** * ***

**Supplemental Figure 2**. Alignment of the 3’ region of M1 and M89 mga DNA sequences. The sequences are from M1 strain MGAS5005 and M89 strain KUN-0012590 (Murase et al., 2020). The c.1571C[8] polycytidine tract in M1 *mga* is highlighted in yellow.
